# A Flood of Microbial Genomes–Do We Need More?

**DOI:** 10.1371/journal.pone.0005831

**Published:** 2009-06-09

**Authors:** Niyaz Ahmed

**Affiliations:** Pathogen Biology Laboratory, Department of Biotechnology, School of Life Sciences, University of Hyderabad, Hyderabad, India; University of California, Berkeley, United States of America

Complete genome sequences of important bacterial pathogens and industrial organisms hold significant consequences and opportunities for human health, industry and the environment. Addressing biological and clinical problems through genome sequence based approaches offers many commercial opportunities. The aftermath of whole genome sequencing has revealed new insights into evolution of bacterial lifestyles including strategies for adaptation to new niches and overcoming competitors. Whole genome sequences representing more than 1500 prokaryotic organisms combined with the dozens (to hundreds) of strain re-sequencing projects are posing mind boggling problems on the optimal utilization of the resultant ‘omic’ datasets. Consequently, microbiologists are confronted with the challenge to translate these data into better human and animal healthcare solutions and pursue basic research approaches to interpret the data in ecological and evolutionary perspectives. New informatic approaches towards optimal utilization, holistic integration and meaningful interpretation of the genome sequence data are extremely necessary.

## Introduction

Whole genome sequence analysis of prokaryotes is fundamentally important in understanding human infections, development of diagnostics and vaccines, biodefense studies, antimicrobial target identification and drug design. Rapid advances in sequencing technology have provided the capability to quickly and cheaply produce several hundreds of prokaryotic genomes each year.

The next generation sequencing platforms (454 from Roche, Solexa of Illumina, and SoLiD from ABI) hold promise to further reduce time and cost of whole genome sequencing. Multiple species of bacteria and hundreds of strains thereof are being sequenced every year, thanks to cutting edge approaches such as re-sequencing wherein genome sequence of a reference organism is used as a scaffold to direct analysis of several different strains [1]. Using this method, multiple whole-genome bacterial sequencing projects can now be completed in less than two weeks instead of months. The total number of completed genomes (including reference and strain re-sequencing projects) is consistently doubling every 16 months [2] by adding about 20 new genomes every month [3]. By the end of March 2009, a total of 1775 prokaryotic genome sequences and draft assemblies were available in the NCBI genome database. At this pace of sequencing output, study of a single bacterial genome has become almost pedestrian while the comparisons of multiple strains of a single species is within the relatively easy reach.

Comparison of genomic sequences has revealed mechanism of changes in bacterial lifestyles. We have learned how species have evolved strategies to survive and compete as part of adaptation to their preferred hosts, habitats or niches. Genomic comparison of multiple species and strains has facilitated insights into adaptive mechanisms leading to host or tissue tropism. Such inferences however need to be tested functionally and thus the need for integration of genome data with cues obtainable from downstream ‘omic’ experiments that have sampled a variety of conditions or treatments. New informatic approaches are emerging which are capable of integrating genomic and functional datasets and also making use of data available through published resources. The emergence of e-Science, Semantic Web, and Science 2.0 approaches hold a lot of promise for holistic data integration and meaningful interpretation of community genomics and microarray experiments in an interactive and collaborative fashion. The present overview discusses some of these issues and ideas in relation to the ‘PLoS ONE prokaryotic genomes collection’.

## Genomic insights - lifestyles, adaptations and pathogenic mechanisms

Comparative genomics of whole genome sequences of many different pathogenic and commensal forms of microorganisms have improved our perception of the mechanisms of pathogenesis and the transition between pathogenic and non-pathogenic varieties within the same species. It is becoming increasingly evident that distinct genomic differences found in different microbes have a definite impact on pathogenic potential, adaptation to parasitic lifestyles and host/tissue tropism. Some examples in this context are discussed.

In the case where different species of the same genus represent diverse lifestyles it is imperative to have sampled genome sequences from varieties of all forms. For example, the availability of three complete genome sequences from *Acinetobacter* (i.e. AYE, SDF and *A. baylyi* ADP1) has enabled comparison in a more general context to tease apart likely genetic changes that enabled adaptation of *Acinetobacter* species to specific environments [4]. While the three organisms share a large chunk of genes, major differences exist in terms of their flexible genome component such as prophages and insertional sequences [4].

Another interesting lifestyle has been deciphered from the genome sequence of *Brachyspira hyodysenteriae* [5], an anaerobic intestinal spirochaete that colonizes niches of swine colon and causes dysentery of pigs, a disease of significant economic importance. It appears that the bacterium may have evolved strategies to survive and adapt via gene transfer in the intestinal environment. The genome sequence data suggests presence of genes encoding anaerobic metabolism and mechanisms to cause mucosal damage through the activity of many different virulence factors facilitated by chemotaxis and motility. Interestingly, the chunks of genes believed to have been horizontally acquired by *Brachyspira*, and that are supposed to have facilitated adaptation and survival of the bacterium within the intestines, belonged mostly to classically ‘enteric type’ of organisms, rather than to other spirochaetal relatives of *Brachyspira* [5].

Whole genome sequencing and analysis of *Mycobacterium indicus pranii* (MIP) together with molecular phylogenetic analyses [6] revealed a unique soil and water dwelling lifestyle for this ‘generalist’ organism. MIP had a common ancestor with pathogenic *Mycobacterium avium intracellulare* complex that did not prefer parasitic adaptation but a free living life-style. Further analysis suggests a shared aquatic phase of MIP with the early pathogenic forms of *Mycobacterium*, well before the latter diverged to form ‘specialist’ bacterial parasites. This information has an important bearing on our understanding of mycobacterial evolution.

Genomic downsizing and streamlining has been a dominant evolutionary trend in mycobacterial genome evolution that perhaps shapes their host-range and tissue tropism giving rise to ‘specialist’ lineages [6], [7]. Another interesting example of genome optimization through reduction - based - metabolic optimization comes from *Yersinia pestis* which originated from its closest relative *Y. pseudotuberculosis* [8]. The same has been true in the case of *Brucella ovis* whose genome is shorter than the classical zoonotic strains [9] oving to loss of genes *via* pseudogenization and degradation that has happened concomitant to the narrowing of its host range; it infects only sheep [10]. It has been suggested that inactivation of genes linked to nutrient acquisition and utilization, cell envelope structure and those encoding urease may have played a role in narrowing of the tissue predilection and host range of *B. ovis* [10]. Another important feature of the *B. ovis* genome has been the presence of increased number of transposable elements thus hinting towards frequent shuffling (genomic fluidity, or plasticity) of its genome [10].

Variation in gene content, especially the flexible or unstable part of the genome such as mobile elements and genomic islands, has been shown to influence phenotypes such as virulence and antimicrobial resistance. This is especially true for some of the biomedically significant organisms such as the Group A *Streptococcus* (GAS). Recently, a study analyzing twelve sequenced GAS genomes [11] determined that the resultant ‘metagenome’ holds tremendous potential for understanding pathobiology of the GAS. This multi-genome dataset provides an opportunity to address putative functions, encoded by the exogenous genetic elements, such as antimicrobial resistance. Another major benefit from these genomes includes the ability to develop molecular markers based on GAS mobile elements to tag and track field-level diversity of the circulating strains; this will be of paramount significance in vaccine development and testing.

## Why sequence multiple species and strains?

A wide variety of microbial sequencing projects having been completed or being implemented throughout the world has created a rich and diverse ‘mega-database’ of microbial genomes. However, to fully gauge the prevailing diversity and stratification patterns of all bacterial species, it will be required to sequence hundreds and thousands of genomes representing all branches and lineages within the bacterial and archaeal part of the tree of life wherein each of the phylum provides an opportunity to capture evolutionary footprints of billions of years. It is estimated that there are at least 35 different phyla of bacteria according to the rRNA gene sequence based tree of life [12]. The genome sequences of bacteria that have accumulated so far represent only three phyla, thus leaving major gaps in the genomic representation of the bacterial diversity of our biosphere. It is therefore urgently required to sequence genomes from underrepresented phyla and to improve resolution of deep branches in the bacterial tree so as to enable biological studies of important lineages and to decipher novel functions thereof. In view of these facts more systematic approaches to the sequencing of the microbial genomes are needed to leverage data for the interpretations of environmental surveys as well as to facilitate comparative genomic analyses and annotations of different genomes and microbiomes. The GEBA (Genomic Encyclopedia of Bacteria and Archaea) project is one such ‘community phylogenomics’ initiative that is being implemented at the Joint Genome Institute (http://www.jgi.doe.gov/programs/GEBA/). This program aims at filling the genomic gaps pertaining to bacterial and archaeal branches of the tree of life while using the tree itself as a guide to identify which target microorganisms need to be sequenced completely. Some of the potential benefits of the GEBA project include identification of new protein families across different lineages of bacterial phyla so as to provide a comparative genomics and proteomics platform towards annotation of forthcoming genomes and microbiomes of the same or different phyla. Also, it will facilitate improved phylogenetic anchoring of metagenomic data-sets besides providing better understanding of the processes underlying the evolutionary diversity and functional stratification of different microbes inhabiting various different niches in the environment.

Many of the pathogenic bacterial species are monomorphic meaning that they present very little diversity upon genetic fingerprinting or limited sequence profiling. Gaining insights into their dispersal patterns, evolutionary genetics, emergence and reemergence in different communities and catchments poses a great challenge for molecular epidemiologists. Multiple genome sequences from across strains of a single species offer more fine scale resolution of genetic differences that enable tracking and identification of species and development of additional genetic markers.

Prokaryotes evolve largely by horizontal gene acquisition, vertical genome reduction and *in-situ* gene duplication strategies to shape an optimal repertoire of the genes and elements to support a successful lifestyle [7]. Lateral gene flow is widespread among different strains of a single species and most bacterial organisms acquire novel functions through harnessing functional attributes of some of the genes gained through such recombinational processes. One important message that has emerged from the analyses of complete genomes is–microbes are diverse and highly adaptable. To know why it is so, we need further insights through individual and community level genomics. Such federated genomics approaches are also likely to help us answer several outstanding questions such as, how virulence evolves as a function of genome optimization under different compulsions offered by a colonized niche; how microbes regulate their genomic streamlining; what environmental stimuli are responsible for the diversification and stratification of microbial lineages; what is the functional significance of prokaryotic genomic diversity especially in the context of host and tissue tropism and towards understanding parasitism versus commensalism; and how can microbial genome data and the observed diversity be experimentally harnessed for the generation and selection of optimally adapted microorganisms? These questions clearly underpin case for sequencing additional representatives from different pathogenic microbial species.

Novel genes constantly emerge from newly sequenced replicate genomes [13], [14] and thus the concept of a ‘dockyard’ of genes (of presumably unknown functions) that each of the strains harbors. This paradigm was supported by the analyses wherein the pan-genome of a true bacterial species is described to be ‘open’ and each new genome sequence would identify dozens of new genes in the existing pan-genome of *Steptococcus agalactiae* for example [14]. It is clear also from previous studies that such pool of strain specific genes in pathogens such as *Helicobacter pylori*, termed the ‘plasticity region cluster’, could be useful in adaptation to a particular host population [15]. This pathogen shows a very strong geographic adaptation and is known for harboring up to 45% strain specific genes with most of them gained through horizontal gene transfers [7], [15]. Recently the members of the plasticity region cluster were shown to be likely involved in promoting proinflammatory potentials of some of the strains thus providing a survival advantage [16], [17].

Another important reason to sequence replicate genomes of a prokaryotic species entails need to study chronological evolution of bacterial pathogens within their hosts. The nature and extent of genetic polymorphisms accumulated in the genome of bacterial pathogens across wide timescales and during the colonization of different host niches are not known. The advantages of polymorphisms linking to fitness in pathogens or commensals need additional in-depth studies. While some studies have explored chronological strain diversity through genetic fingerprinting [18], microarrays [19] and limited sequencing [20], whole genome profiling of isolates obtained at different time points and sampled from different sites is required to investigate the frequency and timing of the emergence of small insertions, deletions and substitutions and their functional significance in terms of adaptive mechanisms.

With complete genomes of multiple variants of a closely related group (genus or species), it is possible to test evolutionary hypotheses based on the core genes of the group. The phylogenetic relatedness of such core genes could then be harnessed to examine larger collection of strains by multilocus sequence typing (MLST). This genome sequence based approach has already revolutionized molecular epidemiology and evolutionary genetics of many bacterial pathogens as previously reviewed [21]. The most noteworthy case is of *Leptospira interrogans* whose genome sequences enabled significant insights into the question as to how virulence evolves during the traverse of pathogens from one intermediate host to the other. This has been facilitated through comparative genomics with saprophytic *L. biflexa* genome sequence [22] as well as genome guided insights into phylogeny of various species of the pathogen [23] and through differences between saprophytic and pathogenic species [22]. Based on the core genome of pathogenic and saprophytic strains, a sensitive and accurate MLST [24] method was developed to track and analyze individual strains of different species at population levels; a task which was otherwise impossible by using traditional serotyping approaches. This is because the serotype is often influenced by frequent lateral gene transfer events within the loci that determine repertoire of cell surface antigens.

Leaving aside genetic diversity of naturally occurring populations, important differences in the isolates of even a single laboratory strain might be highly significant in genetic experiments. Using whole genome sequence determination, several important polymorphisms were detected in replicate genomes of a single strain of *Bacillus subtilis* [25]. Such approaches allow rapid identification and mapping of single nucleotide polymorphisms and mutations linked to different phenotypes because they are less laborious and definitely cheaper than genetic mapping experiments.

## Making sense of the genome piles

Developing the computational infrastructure necessary to support data analysis and formulation of tools and resources is necessary to fully utilize the wealth of genomic information. Novel data integration capabilities in a community genomics environment are likely to give rise to cutting-edge platforms. However, availability of processed data to feed into such platforms will depend on the speed and accuracy with which the genomic raw data and assemblies are processed. It is noteworthy to mention the success of subsystems approaches wherein annotation servers have been developed that are capable of processing 20–50 prokaryotic genomes daily. Such tools as the RAST server [26] can annotate up to 200–300 genomes per month. This machine identifies RNA-encoding and protein-encoding genes, assigns functions to the genes, and attempts to place the genes within genomic subsystems, producing an initial estimate of which subsystems (i.e., pathways, complexes, and non-metabolic components of the cell) are present in the genome. The accuracy of the annotations arises from manual curation of a library of over 800 subsystems that include over 1.5 million genes with functions assigned from a controlled vocabulary.

Processed genomic information as above is likely to make up excellent inputs for the systems that exploit the power of collaborative grid computing aimed at integration of information that links organisms through their genes and gene products *via* a semantic web approach [27]. Bacterial genome experts, microbiologists, evolutionists and clinical research specialists are likely to benefit from tools that could quickly identify and explore genome encoded features that help decipher particular lifestyles, survival advantages, core metabolic pathways, plastic zones, diagnostic markers and drug targets. This of course needs processing and comparisons of multiple datasets in an *in silico* or a ‘virtual’ laboratory [28]. The complexity of such projects however, requires an e-Science approach wherein a computational environment enables transparent and seamless access to distributed datasets, through scientific workflows that automate *in silico* experimentation across grids of international networks [27]. One such revolutionary resource which integrates different forms of federated information comprising of genomic sequences and associated metadata relating to various marine microbial sequencing projects is CAMERA (Community Cyberinfrastructure for Advanced Marine Microbial Ecology Research and Analysis) [29]. This is a highly robust community approach to support a fundamental paradigm shift in the way microbial genomic datasets are analyzed and interpreted. One of the future challenges of such platforms that are focused on the genomic datasets is how they can be integrated with information from functional analyses of transcriptomes, regulomes, proteomes, interactomes and metabolomes of organisms in a dynamic and interactive fashion.

Academic institutions and publicly funded research consortia are generating large sets of ‘omic’ data that are capable of serving collaborative groups across different disciplines. With the cutting-edge approaches as discussed above, it will be possible to facilitate these groups to bring in and compare their sets of data with other experimental results and pathways available in the public domain. Web tools based on these concepts (for example, nextBio; http://www.nextbio.com) routinely extract, integrate and compare information and observations contained in publications while juxtaposing colossal amounts of disparate, biological or clinical and ‘omic’ data from public and proprietary sources, regardless of data type and origin. Other tools such as Ondex [30] display biological data as a set of linked graphs with the nodes representing a data object and the edges representing a relationship between the two nodes (http://ondex.sourceforge.net/).

## PLoS ONE Prokaryotic Genomes Collection

The possibilities and proposals towards computational processing of genome data as discussed appear mind boggling at this stage, but ultimately scientists will be empowered to swiftly interpret their own experimental results within the context of other published research findings in a more interactive and collaborative way. These advances underscore the need for important biological information such as genome sequences and microarray data sets to be made freely available and the literature describing the data interpretation to be available through Open Access platforms such as PLoS ONE. Since PLoS ONE publishes research through extensible markup language (XML), it is possible to quickly exchange experimental results and their interpretations across different platforms. This in turn simplifies utilization and processing of genomic information contained in research publications so that details such as decipherment of novel pathways or evolutionary relationships etc. could be discussed globally and interpreted through community genomics environments.

To this end, ‘PLoS ONE prokaryotic genomes collection’ represents a novel initiative to compile a permanent archive of all important articles describing whole genome sequence based biology of prokaryotic organisms. This collection of articles will facilitate understanding of the biology and lifestyle of the underlying organisms not only through main contents of articles but also *via* information from external sources that discuss and link to the results, such as citations from PubMed Central, Google Scholar and Scopus; evaluations and ratings at Faculty of 1000; bookmarks from social networking sites such as CiteULike and Connotea; and blog posts from experts and readers in the field. Just like other PLoS content, it will be possible to make utilization of individual articles interactive for the users (human or machine) to harness elements of research (annotation tables, phylogenetic trees, evolutionary hierarchies, gene expression data, graphs, texts etc.) and associated content in the form of relevant discussions (and raw data posted in response to a discussion). This content can be processed in a variety of computational formats such as graphs or networks that can be inspected visually, cured manually or mined computationally. Linking therefore the secondary contents and Science 2.0 based enhancements to published information and their subsequent harnessing through different knowledge-platforms is likely to underpin formation of new ideas and insights in a more holistic and interdisciplinary manner. Such novel theses in the form of alternative or even more provocative interpretations could ultimately be linked back to the original genome sequences thus completing a cycle of information sharing through Open Access.
